# *Mycobacterium leprae* Transcriptome During *In Vivo* Growth and *Ex Vivo* Stationary Phases

**DOI:** 10.3389/fcimb.2021.817221

**Published:** 2022-01-12

**Authors:** Olabisi Ojo, Diana L. Williams, Linda B. Adams, Ramanuj Lahiri

**Affiliations:** United States Department of Health and Human Services, Health Resources and Services Administration, Health Systems Bureau, National Hansen's Disease Program, Baton Rouge, LA, United States

**Keywords:** *Mycobacterium leprae*, transcriptome, RNA-Seq, metabolic pathways, mouse footpad

## Abstract

*Mycobacterium leprae*, the causative agent of leprosy, is an obligate intracellular pathogen primarily residing within host macrophages and Schwann cells. Whole genome sequencing predicts a highly degraded genome with approximately one third of the coding capacity resulting in the loss of many catabolic pathways. Therefore, it can be assumed that *M. leprae* obtains many of the necessary metabolites for intracellular survival and growth from the host cells. In this study, global transcriptomic analyses were done on freshly harvested *M. leprae* growing in athymic mouse footpads for five months (MFP5) and compared to those held in axenic medium for 48 (ML48) and 96 (ML96) hours. Results show that all of the genes and pseudogenes were transcribed under both *in vivo* and *in vitro* conditions. 24% and 33% of gene transcript levels were significantly altered in ML48 and ML96 respectively, compared to MFP5. Approximately 45% (39/86) of lipid metabolism genes were significantly downregulated in ML96 compared to MFP5, majority of which are in the β-oxidation pathway. Cholesterol oxidase, acyl-CoA dehydrogenase, and coenzyme F420-dependent oxidoreductase, were significantly upregulated in both ML48 and ML96 compared to MFP5. 30% of cell wall and cell processes functional category genes had altered gene transcription at 96hr compared to MFP5. 40% of 57 genes associated with mycobacterial virulence showed significantly altered transcript levels with 52% significantly downregulated in ML96, including most of the Pro-Glu/Pro-Pro-Glu genes. All 111 hypothetical protein genes with unknown function were expressed. Adenosine triphosphate (ATP) synthesis in *M. leprae* appears to be significantly downregulated under *ex vivo* conditions. This is the first study comparing *M. leprae* global gene expression during *in vivo* growth and *ex vivo* stationery phase in axenic medium confirming that during the growth phase in the footpads of experimentally infected mice, *M. leprae* is metabolically active and its primary source of energy production is probably lipids.

## Introduction

*Mycobacterium leprae*, a gram-positive, acid-fast bacterium and the etiologic agent of leprosy (a.k.a Hansen’s disease, HD), affects the skin, eyes, mucosa of the upper respiratory tract and is unique among bacterial pathogens in its ability to invade peripheral nerves. Currently around 200,000 new leprosy cases are diagnosed each year globally. It is estimated that about 25% of the newly diagnosed patients each year will suffer from irreversible nerve damage and are at risk of developing classic hand or foot deformities and associated disabilities as a long-term consequence of leprosy. Approximately three million individuals around the world suffer from physical disabilities because of damage to peripheral nerves and subsequent sensorimotor loss caused by leprosy. (WHO, 2021).

A key to the success of *M. leprae* as a persistent, obligate intracellular pathogen is its ability to survive and grow for extended periods within professional phagocytes, primarily macrophages, and Schwann cells, as its host. Sustained growth within these cells requires avoiding or resisting antimicrobial mechanisms and adapting to a stressful environment. Our limited understanding of these survival strategies of *M. leprae* during intracellular growth has been mainly through use of the mouse footpad (MFP) infection model (Shepard, 1962; Lenz et al., 2020). In this model, *M. leprae* grows very slowly with a predicted doubling time of around 12 days (Shepard & McRae, 1965). This is in contrast to *M. tuberculosis*, a close relative, which has a doubling time of around 16 hours under optimal conditions, and is able to grow both intracellularly and extracellularly (Beste et al., 2009). Additionally, *M. tuberculosis* is able to grow *in vitro* in minimal media containing a single carbon source such as glycerol, a nitrogen source and a few essential mineral ions (de Carvalho et al., 2010).

*M. leprae* possesses a highly degraded genome resulting in the loss of approximately 1/3 of the coding capacity found in *M. tuberculosis* (Cole et al., 2001; Eiglmeier et al., 2001); yet, it has retained complete enzyme systems for the synthesis of all purines, pyrimidines, nucleosides, nucleotides, together with most amino acids, vitamins and cofactors. Glucose metabolism appears to be intact with the exception of pyruvate metabolism to acetyl CoA. In addition, most anabolic and several catabolic pathways in lipid metabolism are intact. However, redundancy of many of these pathways has been eliminated and many important catabolic pathways are impaired or lacking e.g., the aerobic respiratory chain of *M. leprae* is truncated and anaerobic respiration is defective (Wheeler, 2001). The inability to cultivate *M. leprae* on artificial medium has hindered research into many aspects of *M. leprae* physiology and host-pathogen relationship, which are essential to understand the disease and to develop better clinical interventions. In the present study we determined the gene expression profile of *M. leprae* during its growth phase in the MFP infection model using transcriptome analyses (RNA-Seq) and have demonstrated the ability of *M. leprae* to alter its global gene expression profile in response to its environment (physiological and nutritional stress) in axenic medium. Comparison of functional metabolic networks of intracellular *M. leprae* during the growth phase in the MFP model to that held in axenic medium for up to 96 hours (metabolically active but replication deficient) reflect potential metabolic changes due to these physiological and nutritional stresses and may direct us in developing an artificial medium capable of supporting *M. leprae* growth. To our knowledge, this is the first study to compare the global transcriptome of *M. leprae* under *in vivo* and *ex vivo* conditions.

## Materials and Methods

### Mycobacterium leprae

*M. leprae*, strain Thai-53, is maintained in BALB/c athymic nude mice (Envigo) through serial passage at the National Hansen’s Disease Program (NHDP) laboratory. In the current study, nude mice were infected by inoculating each hind footpad with 3 x 10^7^ *M. leprae*. At 5 months post inoculation mice were euthanized and MFP tissue was removed. MFP tissues were either immediately fixed in 70% ethanol or homogenized for purification of viable *M. leprae* using a previously described protocol (Truman and Krahenbuhl, 2001). Freshly harvested bacteria were adjusted to 1x10^9^/ml in NHDP medium (**Supplementary Table S1**) and incubated at 33°C. At 48 hr and 96 hr post incubation, *M. leprae* viability was analyzed using radiorespirometry (RR) (Franzblau et al., 1992) and viability staining (VS) (Lahiri et al., 2005a). Bacteria were pelleted and fixed in 70% ethanol. Prior to RNA extraction ethanol was removed and *M. leprae* were treated with 0.1N NaOH to remove residual mouse tissues (Lahiri et al., 2005b).

### Radiorespirometry and Viability Staining

The metabolic activity of a suspension of *M. leprae* was determined by measuring palmitic acid oxidation rate as described previously (Franzblau et al., 1992). Briefly, 1x10^7^ *M. leprae* were suspended in 1.0 ml of 7H12B medium with ^14^C-palmitate as the carbon source, in a 6 ml glass shorty vial (Wheaton Industries Inc.) with a loosened cap. The shorty vial was placed into liquid scintillation vial with a strip of Whatman #42 filter paper (Fisher Scientific) soaked in Kodak concentrate I (Eastman Kodak Co.) to capture the ^14^CO_2_. The vials were incubated at 33°C and the cumulative counts per minute (CPM) were determined following 7 days of incubation. Results are calculated as log_10_ CPM ^14^CO_2_ per 10^7^ bacilli and reported as mean +/- S.D.

Viability staining was done using the BacLight Viability Staining Kit (LifeTechnologies) as described previously (Lahiri et al., 2005a). Briefly, 2x10^7^ *M. leprae* were incubated for 15 minutes at room temperature in 6 mM Syto9 and 30 mM propidium iodine. Following staining the bacilli were washed and finally resuspended in 5% glycerol in saline. Five μl of the suspension was spread onto a slide, and viability was determined by counting the red and green bacilli, indicating dead and live bacteria, respectively, under a Nikon fluorescence microscope. Results are calculated as percent viability and reported as mean +/- S.D. Mann-Whitney U test was used to determine significant differences (*P*<0.05) between groups.

### RNA Purification and RNA-Seq

Bacterial lysis and RNA purification was performed using a FastPrep-24 vertical homogenizer (MP BioMedicals) and FastPrep Lysing Matrix B tubes following a previously described protocol (Davis et al., 2013). After DNase treatment, an aliquot of sample was analyzed for the presence of genomic DNA contamination by PCR. Ribosomal RNA was depleted from total RNA preparations using a Ribo-Zero rRNA Removal Kit following the manufacturer’s protocol (Illumina). RNA quantity and quality was determined using NanoDrop 2000 (Thermo Fisher) and Agilent 2100 BioAnalyzer (Agilent Technologies). Sequencing samples were prepared using the SOLiD^®^ Total RNA-Seq Kit (Life Technologies) with 100 ng ribosomal RNA-depleted and barcoded (SOLiD™ RNA Barcoding Kit, Module 1-16, Life Technologies) according to the manufacturer’s protocol. Emulsion PCR and SOLiD sequencing, 75 base pairs single direction, were performed according to manufacturer’s instructions for the SOLiD™ 5500 System (Life Technologies).

### RNA-Seq Data Analysis

Sequencing analysis was done using Bacterial RNA-seq Analysis Kit 1.0 on the Maverix Analytic Platform (Maverix Biomics, Inc, San Mateo, CA). Raw sequencing reads from the SOLiD sequencing platform were converted into FASTQ file format and quality checked for potential sequencing issues and contaminants using FastQC (FastQC). Adapter sequences, primers, and reads with a quality score below 13 were trimmed using fastq-mcf of ea-utils (Aronesty, 2013) and Trimmomatic (Bolger et al., 2014). Reads with a remaining length of < 20 base pairs after trimming were discarded. Pre-processed reads were mapped to the *Mycobacterium leprae* TN genome (Accession #NC_002677.1/AL450380.1) using EDGE-pro (Magoc et al., 2013). Read coverage on forward and reverse strands for genome browser visualization was computed using SAMtools (Li et al., 2009), BEDtools (Quinlan and Hall, 2010), and UCSC Genome Browser utilities (Meyer et al., 2013). Read counts for RefSeq genes generated by EDGE-pro were normalized across all samples and then used for differential expression analysis using DEseq (Anders and Huber, 2010). Significant differentially expressed genes were determined by Wilcoxon test using adjusted *P*-value with a threshold of 0.05. Heat maps are drawn using Morpheus program, (https://software.broadinstitute.org/morpheus). Metabolic Pathway prediction analyses were performed using Kyoto Encyclopedia of Genes and Genomes (KEGG) Pathway Database (Kanehisa and Goto, 2000) (http://www.genome.jp/kegg/pathway.html). The transcriptomic data is available at Sequence Read Archive (SRA identifier SRP345404) and at Gene Expression Omnibus (GEO identifier GSE188532).

## Results

### *M. leprae* RNA

**Figure 1:**
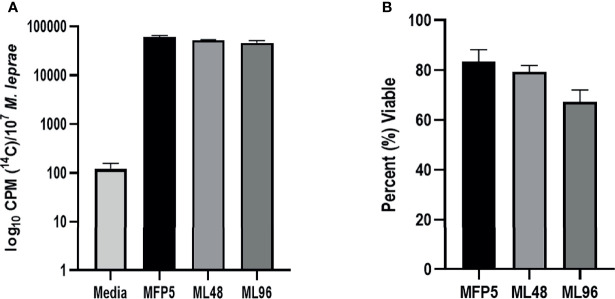
Viability of freshly harvested *M. leprae* from mouse footpad (MFP5) and following incubation at 33°C in NHDP axenic medium for 48 (ML48) and 96 (ML96) hours. Viability measured by **(A)** radiorespirometry and **(B)** syto9/propidium iodide staining. Media alone was used as negative control (Media) for radiorespirometry.

*M. leprae* harvested from MFP tissue at 5 months post infection were free of other microbial contamination and viable. These bacteria maintained their metabolic activity when held in NHDP medium for up to 96 hr, as measured by radiorespirometry (RR) and viability staining (VS). There were no significant differences in RR and VS between the freshly harvested *M. leprae* from MFP (MFP5) and those held in axenic medium for 48 (ML48) or 96 (ML96) hours (**Figures 1A, B**).

Bacterial mRNA-enriched preparations, following rRNA depletion, were obtained from biological replicates of *M. leprae* at these time points. Following trimming, as filtered by adaptors and by quality of read, the average abundance of trimmed reads was aligned to the *M. leprae* genome (TN strain). The average unique reads kept were 29.6 million for MFP5, 34.3 million for ML48 and 30.9 million for ML96. Volcano plots of the overall read abundances of MFP5 and that held in NHDP medium demonstrated that there were significant differences between *in vivo* and *ex vivo*, at both 48 and 96 hours, bacterial gene expression (**Supplementary Figure S1**). However, overall read abundance levels between ML48 and ML96 were not significantly different.

### Expression of Annotated Genes

**Figure 2:**
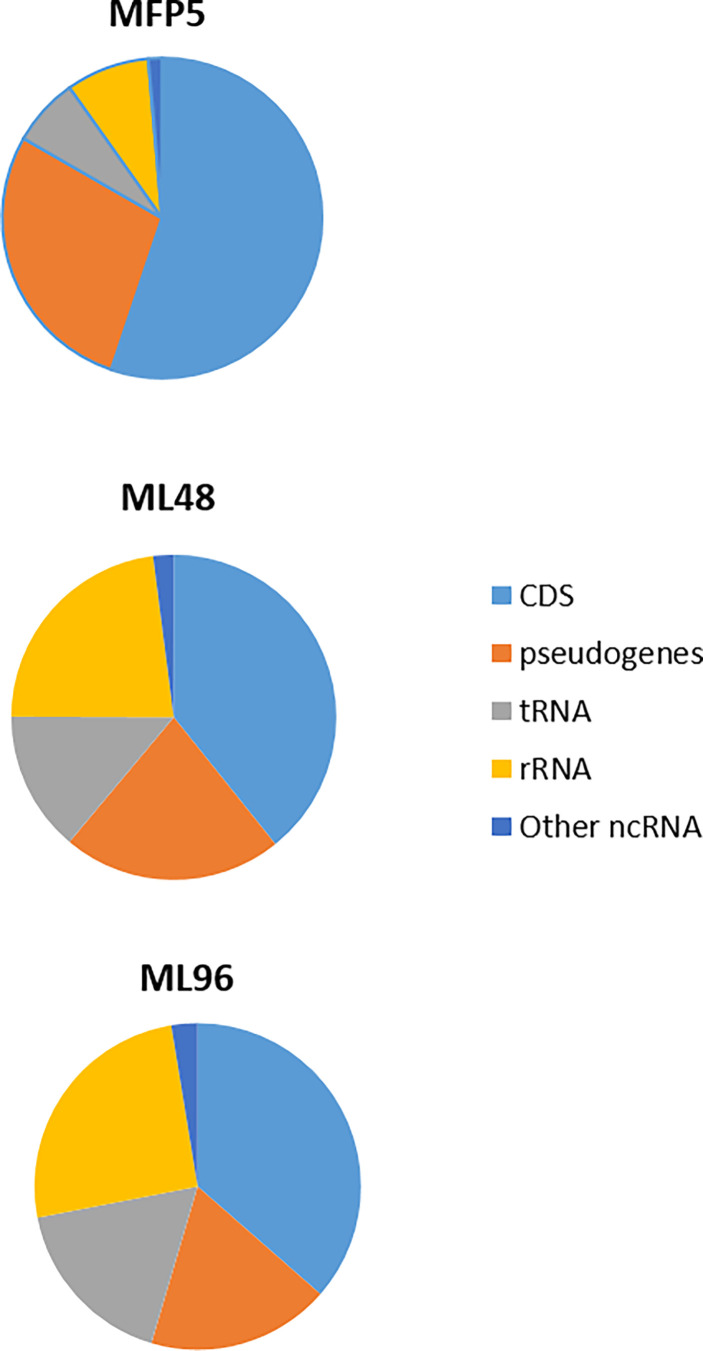
Percentage of transcripts, from MFP5, ML48 and ML96, aligning to coding DNA sequences (CDS), pseudogenes, transfer-RNA (tRNA), ribosomal RNA proteins (rRNA) and other non-coding RNA (ncRNA) of *M. leprae* genome.

Examination of averaged abundance levels of annotated reads demonstrated that all of 2770 genes and pseudogenes were transcribed under all three conditions (GEO identifier GSE188532). The 3 rRNA genes were excluded from further analysis as the samples were depleted for rRNA prior to RNA sequencing. The transcripts included the coding DNA sequences (CDS) of all protein coding genes, including 111 unique “unknown” genes; all annotated pseudogenes; and all 50 stable RNAs. In addition, high levels of noncoding RNAs [tmRNA (ssr, MLS01) and RNase P RNA (MLS02)] were observed (**Figure 2**). All genes with average abundance levels < 500 were either pseudogenes or hypothetical protein genes (GEO identifier GSE188532).

### Comparative Transcription Levels of Open Reading Frames

The average abundance of reads for all genes from samples was normalized using DESeq analysis and grouped according to their functional classification category. Differences in open reading frame (ORF) expression levels were determined between freshly harvested *M. leprae* and those held in NHDP medium in all functional categories except the pseudogenes. Results demonstrated that 24% and 33% of gene transcript levels were significantly altered (*p* ≤ 0.05) in ML48 and ML96, respectively, compared to that of the growth phase in MFP5 (**Table 1**). In addition, ML96 had 149 more genes with altered expression levels than ML48 and included 16% up-regulated genes and 17% down-regulated genes. Out of the total 1122 annotated pseudogenes (1301 according to https://mycobrowser.epfl.ch), 185 had significantly altered expression in ML48 compared to 270 in ML96 when compared to MFP5.

**Table 1:** Transcription of *Mycobacterium leprae* genes with significantly altered expression levels.

Functional Category^a^		Genes with Altered Transcript Levels		Genes with Decreased Transcript Levels		Genes with Increased Transcript Levels	
	MFP	ML48	ML96	ML48	ML96	ML48	ML96
Cell Wall & Cell Processes	380	78	115	48	70	30	45
Conserved Hypothetical proteins	301	68	102	30	50	38	52
Information Pathways	174	38	52	29	35	9	17
Insertion Sequences & Phages	2	0	0	0	0	0	0
Intermediary Metabolism & Respiration	431	85	132	40	63	45	69
Lipid Metabolism	86	25	39	18	30	7	9
PE/PPE Family	11	7	7	7	7	0	0
Regulatory Proteins	56	17	21	5	8	12	13
Stable RNAs	47^b^	44^b^	43	0	0	44	43
Unknown Function	111	18	20	8	10	10	10
Virulence, Detoxification & Adaptation	46	13	16	5	5	8	11
**Total**	1645	393	547	190	278	203	269
**Percentage**	100%	24%	33%	12%	17%	12%	16%
**Pseudogenes**	1122	185	270	79	117	106	153

a Functional Categories: Mycobrowser database http://mycobrowser.epfl.ch/leprosy.html; Accession #AL450380_2.

b Values do not include the 3 rRNAs because of rRNA depletion.

### Comparative Transcription of Genes Associated With Metabolic Pathways

The largest number of differentially expressed genes with known function was observed in the Intermediate Metabolism and Respiration functional category (**Table 1**). Of 132 genes in this functional category with significantly altered gene expression levels in ML96, 48% were down-regulated and 52% were upregulated. Four of the five protein coding genes with the highest level of gene transcription in *M. leprae* during the growth phase in the MFP were from this category.

### Glucose Metabolism

**Figure 3:**
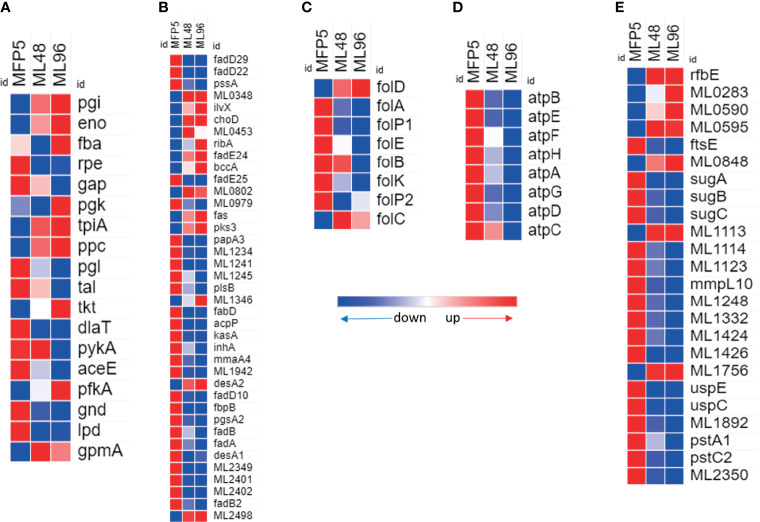
Differential expression (average abundance) of *M. leprae* genes under *in vivo* (MFP5) and *in vitro* (ML48 & ML96) conditions. **(A)** glucose metabolism (https://www.genome.jp/pathway/mle00010), **(B)** lipid metabolism (fatty acid biosynthesis - https://www.genome.jp/pathway/mle00061; fatty acid degradation - https://www.genome.jp/pathway/mle00071), **(C)** Folate biosynthesis (https://www.genome.jp/pathway/mle00790), **(D)** ATP biosynthesis and **(E)** transporter proteins.

Gene transcripts were detected for all genes in the glycolytic and pentose phosphate pathways for glucose utilization in MFP5. Comparative analysis demonstrated the levels of the majority of these gene transcripts were not significantly altered between MFP5 and that of *M. leprae* held in axenic medium, where glucose was supplied as a major carbon source (**Figure 3A**). Expression levels of the majority of genes associated with pyruvate conversion to acetyl-CoA were down-regulated in *M. leprae* axenic cultures including significant downregulation of both *lpd* (ML2387) encoding the dihyrolipoamide dehydrogenase and *dlaT* (ML0861c), encoding the dihyrolipoamide acetyltransferase. In addition, up-regulation of gene transcripts under axenic conditions were detected for *ppc* (ML0578), encoding a unique phosphoenolpyruvate carboxylase not found in *M. tuberculosis*.

### Lipid Metabolism

A total of 39 genes, out of 86, associated with lipid metabolism had significantly (*p*<0.05) altered transcription levels in *M. leprae* held in axenic medium for 96 hours (ML96) compared to that of freshly harvested (MFP5) (**Table 1**). Approximately 76% of these altered genes were down regulated in axenic cultures, where the major carbon source was glucose (**Figure 3B**). The vast majority of these down-regulated genes were associated with the β-oxidation pathway for fatty acid metabolism. Cholesterol oxidase (*choD*, ML0389) along with acyl-CoA dehydrogenase (*fadE24*, ML0661c) and coenzyme F420-dependent oxidoreductase (ML0348) were significantly upregulated during axenic incubation at both 48 and 96 hrs compared to MFP5. Both cholesterol dehydrogenase (ML1942) and 3-hydroxyisobutyryl-CoA hydrolase (ML2401) were also significantly downregulated, under the same conditions.

### Folate Biosynthesis

Transcripts of genes encoding enzymes essential for *de novo* synthesis of folate and its intermediates were detected in *M. leprae* in MFP tissues as well as those held in axenic medium. However, there was a significant reduction in the expression of critical genes in this pathway in *ex vivo M. leprae* including *folP1* (ML0224), encoding the dihydropteroate synthase, and *folA* (ML1518c), encoding the dihydrofolate reductase (**Figure 3C**). Conversely, expression of *folD* (ML0674) was significantly upregulated in axenic medium. The only other gene, in folate biosynthetic pathway, that showed upregulation though not significant was *folC* (ML1471c).

### ATP Synthases

All annotated ATP synthase subunit genes were down regulated in *M. leprae* as early as 48hr *ex vivo*. Seven of the eight genes were significantly down regulated by 96 hr (**Figure 3D**) indicating impaired ATP synthesis in *M. leprae* under axenic condition.

### Transporters

24 genes for probable or putative transporter proteins had significantly altered expression between MFP5 and ML96. 17 of these 24 genes were down regulated at ML96 while the remaining had increased expression when compared to MFP5 (**Figure 3E**). Many of the sugar transporters like *sugA* (ML1087), *sugB* (ML1088) and *sugC* (ML1089) were down regulated in ML96 along with *uspE* (ML1769), *uspC* (ML1770) and *mmpL10* (ML1231). ML0283, a possible cation-efflux transporter was significantly upregulated at 96 hr in axenic medium along with multiple ATP-binding cassette (ABC) transporters like *rfbE* (ML0114c), ML0595, ML0848 and ML1113c.

### Amino Acid Metabolism

**Figure 4:**
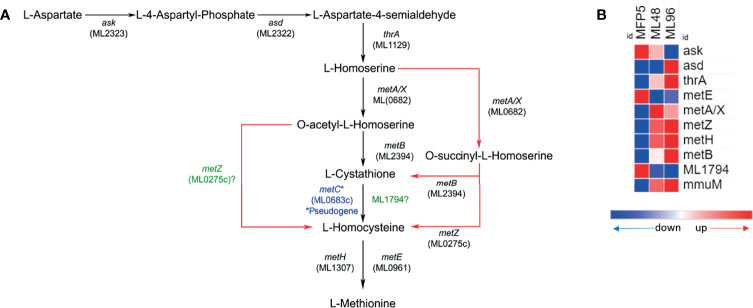
Potential alternate (red arrows) methionine biosynthesis pathway (adopted from KEGG pathway database - https://www.genome.jp/pathway/mle00270) in *M. leprae* **(A)** and expression levels (average abundance) of methionine biosynthesis pathway protein genes in *M. leprae* under *in vivo* (MFP5) and *in vitro* (ML48 & ML96) conditions **(B)**.

The gene expression profile of *M. leprae* in the MFP demonstrated that genes associated with amino acid biosynthesis pathways are transcriptionally active and quite similar to that held in axenic medium. Results of an *in silico* analysis of possible methionine biosynthesis pathways using KEGG, predicted alternative pathways which do not require a functional MetC enzyme. The alternate pathway is depicted in **Figure 4A**. Genes encoding enzymes of this alternative pathway including; *ask* (ML2323c), *asd* (ML2322c), *thrA* (ML1129), *metB* (ML2394c), *metA/X* (ML0682c), *metZ* (ML0275c), *metE* (ML0961c) and *metH* (ML1307) were transcriptionally active in *M. leprae* under all conditions tested (**Figure 4B**). All the genes of this probable alternate pathway were upregulated under axenic conditions with the exception of *ask*, *metE* and ML1794.

### Cell Wall and Cell Processes

**Figure 5:**
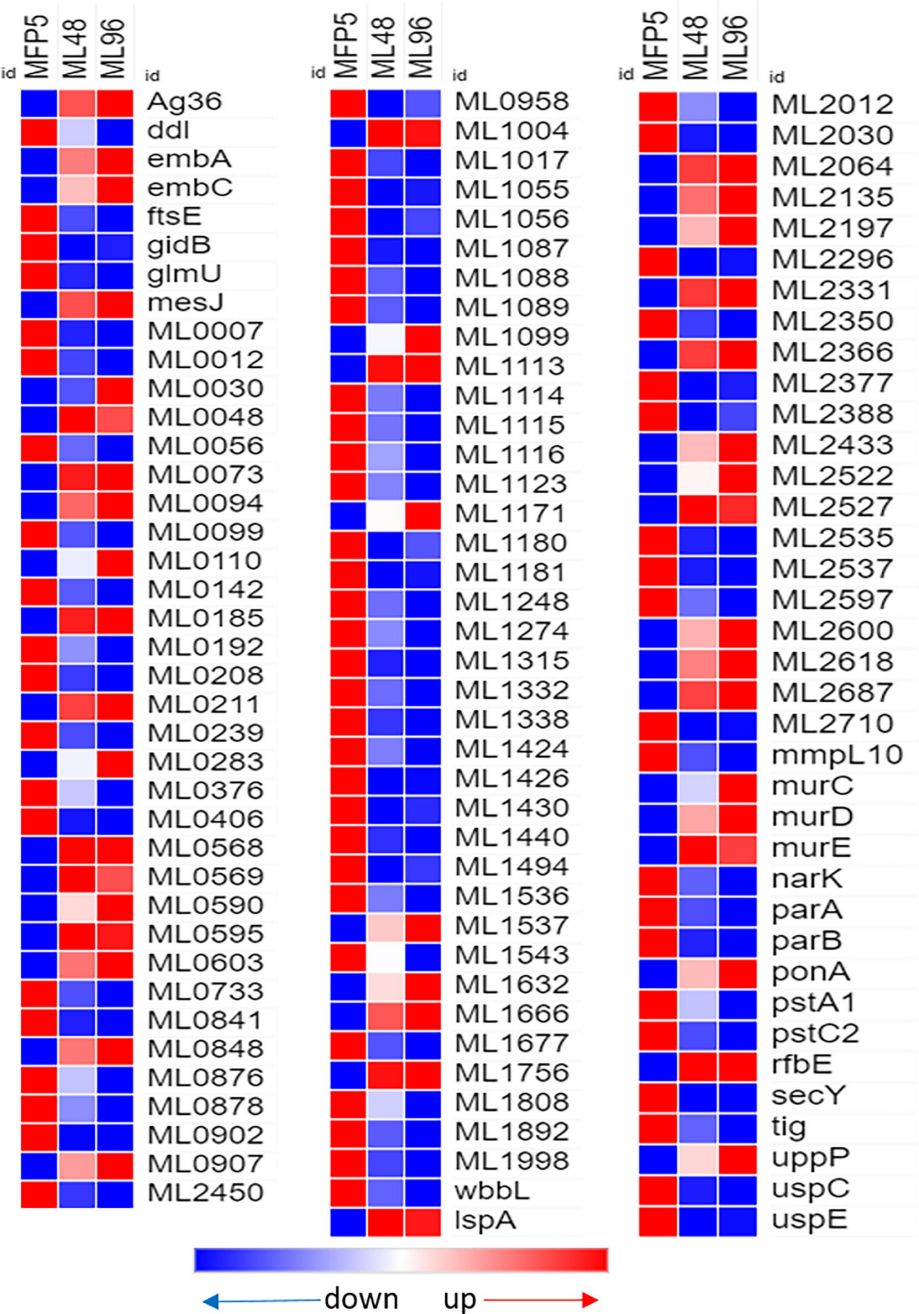
Differential expression (average abundance) of *M. leprae* genes, considered to be in the cell wall and cell wall processes functional category, under *in vivo* (MFP5) and *in vitro* (ML48 & ML96) conditions. Peptidoglycan biosynthesis - https://www.genome.jp/pathway/mle00550, Lipoarabinomannan (LAM) biosynthesis - https://www.genome.jp/pathway/mle00571, Arabinogalactan biosynthesis - https://www.genome.jp/pathway/mle00572.

Out of 380 genes in the Cell Wall and Cell Processes functional category, 30% had altered gene transcription in *M. leprae* held for 96hr in NHDP medium and around 40% of these genes with altered expression had significantly higher gene transcript levels than that of MFP5 (**Table 1**). These included the *murC* (ML0915), *murE* (ML0909) and *murD* (ML0912) genes, encoding proteins involved with peptidoglycan biosynthesis, and *embA* (ML0105c) and *embC* (ML0106c) genes encoding arabinosyltransferases (**Figure 5**). Three genes with the highest fold change in this functional category were hypothetical proteins ML2522, ML0568 and ML0110. In addition, 61% of genes with altered transcript levels in this functional category had significantly lower levels of transcription. There were no significant differences in the expression levels of *murA* (ML1150) and *murB* (ML2447c) between MFP5 and ML48 or ML96.

### tRNAs and Ribosomal Protein Genes

**Figure 6:**
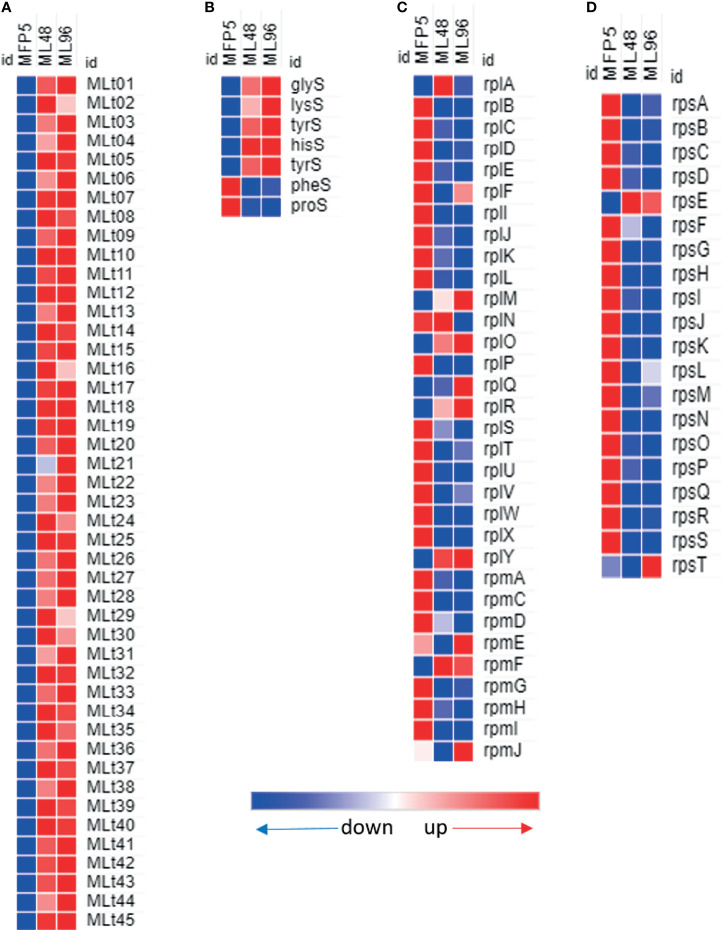
Differential expression (average abundance) of *M. leprae* genes under *in vivo* (MFP5) and *in vitro* (ML48 & ML96) conditions. **(A)** transfer RNA, **(B)** transfer RNA synthetases, **(C)** 50S ribosomal proteins, **(D)** 30S ribosomal proteins.

Increased levels of all of the 45 tRNAs were found in *M. leprae* held in axenic medium and 40 (89%) of these had significantly higher levels of transcription in ML96 than that in MFP5 (**Figure 6A**). Several RNA synthases were also upregulated in axenic medium (**Figure 6B**). In contrast, almost all of the 50S and 30S ribosomal protein genes were down regulated in ML96 compared to MFP5 and around 50% of these down regulation were significant as early as 48hr in NHDP medium (**Figures 6C, D**).

### Virulence and PE/PPE Genes

**Figure 7:**
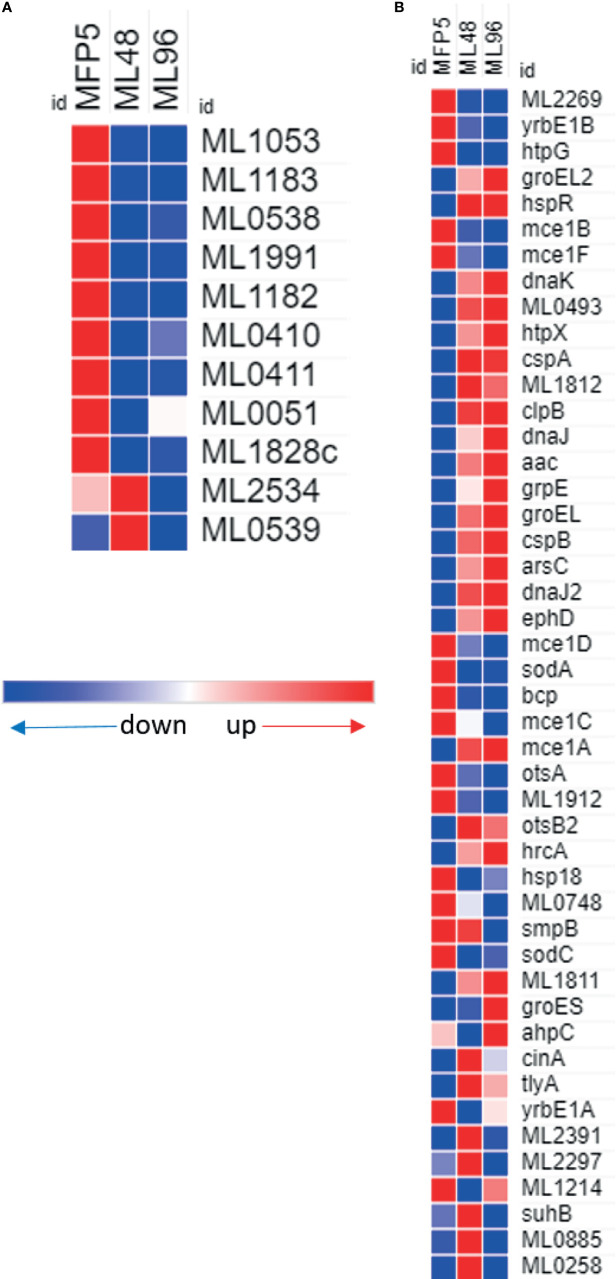
Differential expression (average abundance) of *M. leprae* genes under *in vivo* (MFP5) and *in vitro* (ML48 & ML96) conditions. **(A)** PE/PPE proteins, **(B)** proteins associated with virulance.

A comparison of gene transcript levels of 57 genes associated with mycobacterial virulence demonstrated that 40% of these genes had significantly altered transcript levels (**Table 1**). Of these, 52% were significantly downregulated in ML96, including 7 out of 11 Pro-Glu/Pro-Pro-Glu (PE/PPE) genes (**Figure 7A**). Significantly higher levels of gene transcripts were detected under *ex vivo* conditions for the majority of stress response proteins and regulators like *dnaK* (ML2396c)*, grpE* (ML2495c)*, dnaJ* (ML2494c)*, hspR* (ML2493c), *htpX* (ML2278), *cspA* (ML0198), *clpB* (ML2490c), and *groEL2* (ML0317) (**Figure 7B**).

### Unknowns and Pseudogenes

All 111 *M. leprae* hypothetical protein genes with unknown function were expressed (**Table 1**). Of these, 20 genes had significantly altered gene transcript levels under *ex vivo* conditions as compared to that of MFP5. Approximately 50% had increased levels of transcription while the other half had decreased levels. Protein-Protein Blast results demonstrated that 12 of these protein genes had homologs in other mycobacterial genomes including *M. lepromatosis*, *M. haemophilum* and *M. intracellulare* (**Supplementary Table S2**).

Of the 1000 genes with the lowest expression levels in MFP5, 71% were either pseudogenes or hypothetical proteins. However, pseudogenes and hypothetical protein genes were also found in the moderately and highly expressed groups. Comparative analysis of gene transcript levels in MFP5, ML48 and ML96 demonstrated that 24% of pseudogenes had significantly (*p*<0.05) altered expression levels. Of these pseudogenes, 54% had significantly lower transcript levels in axenic *M. leprae* than in the MFP tissues while 46% had higher transcript levels.

## Discussion

The current study determined the transcriptome of *M. leprae* during growth within the MFP tissues of the immuno-compromised athymic mouse, a model for the lepromatous end of the leprosy disease spectrum (Colston and Hilson, 1976). At 5 months post inoculation, bacteria were highly viable, metabolically active and appeared to utilize their entire protein coding capacity to survive and replicate within the intracellular environment. When *M. leprae* was removed from its intracellular niche and placed in NHDP medium, containing glucose as the main carbon source, dramatic changes in global gene expressions were observed within the first 48 hour. The longer *M. leprae* remained in this *ex vivo* environment, the more dramatic these transcriptional changes became. Gene expression levels were significantly altered in all functional families and included many major metabolic processes. These transcriptional differences between the *in vivo* and *ex vivo* environments likely reflects physiological stresses and potential nutritional deficiencies.

One of the main sources of energy production in prokaryotes is glucose. Genomics predicts that glucose but not acetate and galactose can be used as a carbon source in *M. leprae* (Wheeler, 2001). The current study provides evidence that *M. leprae* has active glucose metabolism, through glycolytic and pentose phosphate pathways, *in vivo* and *in vitro* where glucose is the major source of carbohydrates. However, glycolytic genes transcript levels were not significantly altered (increased) when glucose was supplied as the major carbon source. These results may indicate that glucose utilization as a carbon source for energy production is limited in *M. leprae*. This may be the result of a defect in pyruvate metabolism, which appears to uncouple glycolysis from the tricarboxylic acid (TCA) cycle due to pseudogenization of *pdhA* (ML0441), *pdhB* (ML0442) and *pdhC* (ML0443) genes encoding the E1 and E2 components of the pyruvate dehydrogenase holoenzyme complex (PDhC). The inactivation of the above genes appears to have created a “choke-point” for conversion of pyruvate to acetyl-CoA. Although it cannot be ruled out that *M. leprae* may convert small amounts of pyruvate to acetyl-CoA using the gene products of *aceE* (ML1651), pyruvate dehydrogenase E1 component and *lpd* (dihydrolipoamide dehydrogenase, ML2387), the latter would appear to be less efficient than a fully functional PDhC containing an active dihydrolipoamide aceylytransferase. It is possible that *M. leprae* utilizes glucose primarily for biosynthetic processes or to replenish oxaloacetate (OAA) in TCA cycle by the phosphoenolpyruvate carboxylase (PEPCase) enzyme converting phosphoenolpyruvate (PEP) to OAA. In contrast to *M. tuberculosis, M. leprae* possesses a *ppc* (ML0578) gene, encoding a probable PEPCase. PEPCase plays a role in some bacteria, supplying OAA to the TCA cycle, which requires continuous input of C4 molecules in order to replenish the intermediates removed for amino acid biosynthesis (Eikmanns et al., 1989). The *ppc* gene is transcriptionally active in *M. leprae* under all the tested conditions and the expression was not significantly altered between MFP5, ML48 and ML96. These data suggest that *M. leprae* may be capable of converting PEP to OAA resulting in a mechanism to control pyruvate accumulation and couple glycolysis to the citric acid cycle *via* this OAA intermediate. Furthermore, *M. leprae* appears to retain a fully functional gluconeogenesis pathway signifying the fact that glucose is needed as a central metabolite.

It is likely that *M. leprae* rely on gluconeogenic carbon sources such as fatty acids and amino acids for growth and maintenance. Earlier observations demonstrated that *M. leprae* utilizes long chain fatty acids for production of acetyl-CoA *via* the fatty acid β-oxidation pathway (Franzblau, 1988). Therefore, it was not surprising that the vast majority of genes encoding enzymes for fatty acid β-oxidation pathway were down regulated in NHDP medium, reflecting the lack of lipids in this medium. Together these data support that lipids would be an essential carbon source for axenic growth of *M. leprae*. Among all the differentially expressed genes in lipid metabolism *fadD29* (ML0132) and *fadD2*2 (ML0134), both required for phenolic glycolipid (PGL) biosynthesis in *M. tuberculosis* (Siméone et al., 2010), were highly downregulated under axenic culture conditions compared to MFP5. However, *fadE24* (ML0661), assumed to be involved in fatty acid recycling (Wilson et al., 1999) was slightly upregulated in axenic cultures. ML1654 another gene involved in mycolic acid biosynthesis was also downregulated *ex vivo*. Fatty acid synthase (*fas*, ML1191) on the other hand was slightly upregulated, although *fabG* (ML2565) was not. ML0726 (*bccA*) involved in long chain fatty acid synthesis was also significantly upregulated under axenic conditions. It is also interesting to note that *desA2* (ML1952) thought to be involved in the conversion of saturated fatty acids to unsaturated fatty acids was also significantly upregulated at both 48- and 96 hrs *in vitro* time points. At the same time *desA1* (ML2185) catalyzing similar reaction was slightly down regulated. Taken together these data suggests that in NHDP medium, which is a poor source of lipid, *M. leprae*, may be trying to limit lipid degradation and somewhat enhance fatty acid synthesis and utilization. A recent report suggests that cholesterol may be an essential requirement for *M. leprae* survival and that ML1942 but not *choD* (ML0389) plays a crucial role in the assimilation of cholesterol (Marques et al., 2015; Rosa et al., 2021). Our data shows significant upregulation in the expression of both *choD* and ML1942 during incubation of *M. leprae* in axenic medium for 96hrs, a cholesterol poor environment, compared to that of MFP5.

In prokaryotes, folates serve as essential cofactors in the transfer of one-carbon groups in pathways for the synthesis of methionine, *N*-formylmethionyl-tRNA, glycine, serine, pantothenate, purines, and thymidine (Wheeler, 1992). Many bacterial species, including *M. leprae*, are unable to acquire folates from the external environment and rely on *de novo* folate synthesis to support one-carbon metabolism (Prabhakaran, 1986). Indeed, anti-folate drugs, such as dapsone, have been widely used as an anti-leprosy drug for decades, further suggesting that *M. leprae* cannot utilize host cell-derived folate (Williams et al., 2000). In the current study, a significant reduction in the expression of two essential genes in the folate biosynthesis pathway, *folP1* (ML0224), encoding the dihydropteroate synthase, and *folA* (ML1518), encoding tetrahydrofolate dehydrogenase, was observed. This suggests that while folate production appears to be functional in *M. leprae* within both environments, it may be somewhat compromised in NHDP medium.

The current study demonstrated that all genes associated with amino acid biosynthesis are transcriptionally active during growth in the MFP tissues. Genomics has previously predicted that *M. leprae* is able to synthesize all amino acids *de novo* with the exception of methionine (Cole et al., 2001; Wheeler, 2001). The inability to produce methionine has been attributed to the pseudogenization of *metC* (ML0683c), encoding O-acetyl-L-homoserine sulfhydrylase catalyzing the conversion of L-cystathione to L-homocysteine (Wheeler, 2001). However, revisiting methionine biosynthesis using KEGG pathway tools revealed that *de novo* synthesis of methionine might be possible using cystathionine γ-synthase encoded by *metB* (ML2394c). The same reaction may also be catalyzed by *metZ* (ML0275c) a O-succinyl-L-homoserine sulfhydrylase (Ferla and Patrick, 2014). Interestingly, there is also the possibility of ML1794 catalyzing the l-cystathione to l-homocysteine reaction (https://www.uniprot.org/uniprot/Q9CBM9). All genes encoding enzymes for this pathway are present in the genome and are transcriptionally active in *M. leprae*. There is a strong possibility that *M. leprae* can synthesizes methionine *de novo* using either an alternative methionine pathway or ML1794 and further biochemical studies are needed to confirm the status of methionine biosynthesis in *M. leprae*. Moreover, there may be a need to revisit the annotation of ML0682 to better ascertain whether it is a *metA* or *metX* (Bastard et al., 2017). Transcriptional and pathway analyses of the lysine biosynthetic process in *M. leprae* demonstrated that, while all the genes in this pathway are transcriptionally active, *dapB* (ML1527) and *dapC* (ML2163), encoding the dihydrodipicolinate reductase and N-succinyldiaminopimelate aminotransferase, respectively, are pseudogenes. *In silico* translation of these pseudogenes demonstrated that *dapB* is a translational termination deficient-mutant (lacks an in-frame translational stop codon), resulting in the addition of amino acids to the C-terminus of the DapB protein, which could potentially affect its function. On the other hand, *dapC*, is a highly degraded pseudogene containing multiple translational stop codons within each frame of the sequence. Since there does not appear to be an alternative pathway for lysine biosynthesis, without functional DapB and DapC proteins the lysine biosynthesis pathway appears to be truncated in *M. leprae* possibly making it a lysine auxotroph.

ATP synthase, the enzyme responsible for ATP synthesis, is a multi-subunit complex consisting of a membrane-embedded F0 part (ab2c10-15 subunits) and a cytosolic F1 moiety (α_3_β_3_γδϵ subunits) (Boyer, 2002). This enzyme utilizes the proton-motive force across the bacterial cytoplasmic membrane for the synthesis of ATP from ADP. Even though genes encoding all of the subunits of the ATP synthase complex were transcribed in *M. leprae*, transcript levels for all of the eight ATP synthase genes were lower in axenic medium. Lower quantities of ATP synthase may lead to lower levels of ATP produced in this environment. It has been previously demonstrated that ATP levels decrease over time when *M. leprae* is held in axenic medium (Franzblau and Harris, 1988). Lack of ATP synthesis may be a contributing factor to *M. leprae*’s inability for prolonged survival or growth *in vitro*.

Another important aspect of mycobacterial survival is the composition of its cell envelope. The mycobacterial cell wall consists of a complex array of lipids, glycolipids, proteins and polymers, of which the mycolyl-arabinogalactan-peptidoglycan complex is the major structural component (Mahapatra et al., 2008; Jankute et al., 2015). Peptidoglycan (PG) forms the backbone of the mycobacterial cell envelope, and maintains cell shape and size. *M. leprae* genes involved in PG synthesis and that encoding arabinosyltransferases were all transcriptionally active in *M. leprae* during the growth phase in MFP. However, when *M. leprae* was held in axenic medium, transcript levels of *murD* (ML0912*), ftsZ* (ML0913), *murC* (ML0915), *murE* (ML0909), and arabinosyltransferases encoded by *embA* (ML0105c) and *embC* (ML0106c) were significantly increased. This may indicate an increased effort by *M. leprae* to maintain its cell wall integrity in the axenic environment.

An alternative strategy developed by some bacteria to adapt to changes in their environment is to alter the properties of their cellular envelope through aminoacylation of the membrane lipid phosphatidylglycerol, a component of the lipid bilayer. This process requires the activity of the integral membrane protein, aa-phosphatidylglycerol synthase (aaPGS), to transfer the amino acid (aa) of aa-tRNA onto the phosphatidylglycerol of the membrane (Dare and Ibba, 2012). This has been described in *M. tuberculosis* as a mechanism to alter the properties of its cell membrane in response to cationic antimicrobial peptides. This mechanism requires Lys-tRNA and the two-domain LysX protein (aaPGS) for production of lysinylated PG (Maloney et al., 2009). The *M. leprae* LysX, encoded by *lysX* (ML1393c), is 82.2% identical to that of *M. tuberculosis* and *lysX* is significantly upregulated during incubation of *M. leprae* in NHDP medium. In addition, transcription levels of the vast majority of tRNAs including Lys-tRNA (MLt11) were also significantly increased in axenic environment even though, 41% of ribosomal protein genes had significantly lower transcription levels. This increased transcriptional activity of *lysX* and lys-tRNA genes may support a mechanism to modulate the cell membrane due to environmental stresses in axenic medium. Alternatively, it cannot be ruled out that these tRNAs are accumulating under *in vitro* conditions due to ribosomal stalling (Janssen and Hayes, 2012) which would also result in increased expression levels of ribosomal rescue genes such as tmRNA (ssr, MLS01) and RNase P RNA (MLS02) seen in *M. leprae* as early as 48hr following incubation in axenic medium. Another possible explanation for tRNA accumulation can be due to downregulation of overall translational activities in response to nutritional deficiencies and subsequent bacterial stress response as found in *Chlamydia* (Brinkworth et al., 2018). It is noteworthy that unlike in *Chlamydia*, many of the ABC transporters were upregulated in nutritionally restricted *M. leprae* (ML96).

All the genes associated with mycobacterial virulence, adaptation and detoxification and PE/PPE functional categories were transcribed in *M. leprae*. Approximately 52% of genes with altered transcript levels had significantly lower transcript levels in *M. leprae* held in axenic medium. These included two genes within the *mce1* locus (*mce1B* and *mce1F*). The gene products of this locus are associated with the infection process and maintenance of a productive infection in *M. tuberculosis* (Rengarajan et al., 2005). In addition, transcript levels for the majority of genes belonging to the PE/PPE family were significantly down regulated in *M. leprae* under *ex vivo* conditions. The PE/PPE gene families are uniquely present in pathogenic species of mycobacteria and have been suspected to play a crucial role in the intracellular lifestyle of these mycobacteria (Fishbein et al., 2015). *M. tuberculosis*, also exhibits variable gene expression patterns for PE/PPE genes as it encounters various environmental changes. These data suggest that while *M. leprae* may need these gene products during an active infection, there is less requirement for them *ex vivo*. In contrast, the majority of stress response proteins and regulators had significantly higher gene transcript levels under *ex vivo* conditions including: *dnaK* (ML2396c)*, grpE* (ML2495c)*, dnaJ* (ML2494c)*, hspR* (ML2493c), *htpX* (ML2278), *cspA* (ML0198), *clpB* (ML2490c)*, groEL1* (ML0317) and *groEL2* (ML038). This may not be surprising as *M. leprae* is probably under increased environmental stress when held axenically.

The majority of protein sequences of *M. leprae* have been inferred from computational analysis of its genome and annotated using various bioinformatics tools such as domain homology searches and comparative genomics. 111 hypothetical proteins have not been assigned a functional role nor do they have paralogs in any other species. However, these “Unknowns” may perform a range of biological functions, which may include structural proteins, enzymes or transporters of materials within and between cells, and thereby play a crucial role in the survival, viability and fitness of *M. leprae* in different environments (Mazandu and Mulder, 2012). The current study demonstrated that all “Unknown” genes were expressed in both *in vivo* and *ex vivo M. leprae.* To potentially define a functional role for some of these expressed Unknown proteins, protein-protein BLAST analysis was conducted and identified homologs for 12 of these proteins. Eight of these were found to have homologs in the highly related genomes of *M. lepromatosis* and *M. haemophilum.* The majority of these represented hypothetical proteins in these mycobacterial species. We found that several other homologs were found in multiple mycobacterial species. Interestingly, the Unknown with the highest level of differential expression (5-fold increase) in *M. leprae* held in axenic medium was apparently a truncated homolog of IS481 family of mycobacterial transposases found in multiple mycobacteria including *M. marinium* (67% amino acid identity). The precise function and importance of these Unknown proteins remains to be determined.

Although expression of pseudogenes in *M. leprae* has been described previously (Williams et al., 2009), the present study demonstrated that all annotated pseudogenes are expressed. As a functional category pseudogenes contained the largest percentage of low abundance transcripts in *M. leprae*, they also represented ~25% of the most abundantly transcribed genes. Some of the possible factors responsible for the high rates of pseudogene expression in *M. leprae* are co-orientation of transcribed pseudogenes with transcribed ORFs within or exclusive of operon-like structures and the paucity of intrinsic stem-loop transcriptional terminators between transcribed ORFs and downstream pseudogenes. Previous *in silico* analysis also predicted presence of possible pseudogene promoters (Williams et al., 2004; Akama et al., 2010). The pseudogene with the highest transcription level during growth in the MFP was a highly degraded pseudogene (ML2373), with no known paralogs in the *M. leprae* genome. However, it has a homolog in *M. tuberculosis* (Rv0448c) which encodes a hypothetical protein with unknown function. It is also nonessential for growth in mycobacterial medium or *in vivo* in mice for *M. tuberculosis*. The pseudogene with the highest expression under axenic conditions is ML1049c. This is also a highly degraded pseudogene. It possesses a transcriptional start codon and a prokaryote promoter-like structure 123bp upstream of this transcription start codon (data not shown).

The increased transcript levels of pseudogenes is most likely not due to transcriptional read-through but due to an active promoter. Previous reports have also demonstrated mechanisms for translational “silencing” of many pseudogene transcripts in *M. leprae*, which include the lack of both translational start codons and strong Shine-Dalgarno (SD) sequences (Muro et al., 2011). Transcribed pseudogenes also contain multiple “in-frame” translational stop codons and high Ka/Ks ratios (Williams et al., 2004). This suggests the possibility that although transcribed pseudogenes are much less efficiently translated. Whether pseudogene transcripts have any functional role in *M. leprae* is unclear. Perhaps some pseudogene transcripts have a regulatory role in the expression of their active gene paralogs in the *M. leprae* genome in a similar manner to what has been demonstrated for some human pseudogenes (Pink et al., 2011).

In summary, this study only defines *M. leprae* transcriptome under two different conditions but further studies at the protein expression and metabolite levels are needed to fully understand the physiology of this fastidious organism. Nonetheless, the global transcriptome of *M. leprae* during the growth phase in MFP model indicates that *M. leprae* utilizes all of its ORFs for survival and growth in this environment. This was expected since *M. leprae* has such a degraded genome resulting in the deletion of many alternative pathway genes. This appears to have resulted in a minimal gene set for its very slow growth and replication in the permissive host environment. There is also the possibility that this pathogen is a lysine, and may not be a methionine, auxotroph. Together these analyses also confirm that during the growth phase in the footpads of experimentally infected mice, *M. leprae* is metabolically active and its primary source of energy production is probably lipids. However, both glucose and lipids most likely are needed to be supplemented for axenic growth of *M. leprae*.

## Data Availability Statement

The datasets presented in this study can be found in online repositories. The names of the repository/repositories and accession number(s) can be found in the article/**Supplementary Material**.

## Ethics Statement

The animal experiments were performed in accordance with the United States Public Health Service Policy on the Humane Care and Use of Laboratory Animals. The NHDP Institutional Animal Care and Use Committee (assurance no. D16-00019 [A3032-01]) reviewed and approved all protocols and experiments were conducted in accordance with *The Guide to Care and Use of Laboratory Animals, Eighth Edition.*

## Author Contributions

OO performed all the experimental work. OO, DW, LA and RL performed data analyses. DW, LA, and RL conceived the study and secured funding. DW and RL wrote the manuscript, prepared the figures and tables. Data curation was done by OO and RL. All the authors provided critical reviews and approved the submitted version.

## Funding

The work was supported by the New York Community Trust (Heiser Program) grant No. P12-000281 and in part by American Leprosy Mission grants. Provision of viable *M. leprae* for this study was funded by NIH/NIAID through the Interagency Agreement No. AAI20009-001-00000 with HRSA/HSB/NHDPNIH/NIAID IAA.

## Author Disclaimer

The views expressed in this article are solely the opinions of the authors and do not necessarily reflect the official policies of the U. S. Department of Health and Human Services or the Health Resources and Services Administration, nor does mention of the department or agency names imply endorsement by the U.S. Government.

## Conflict of Interest

The authors declare that the research was conducted in the absence of any commercial or financial relationships that could be construed as a potential conflict of interest.

## Publisher’s Note

All claims expressed in this article are solely those of the authors and do not necessarily represent those of their affiliated organizations, or those of the publisher, the editors and the reviewers. Any product that may be evaluated in this article, or claim that may be made by its manufacturer, is not guaranteed or endorsed by the publisher.

## Supplementary Material

Supplementary Figure S1 Volcano plot showing significant changes in gene expression in ML48 and ML96 when compared to freshly harvested *M. leprae* from mouse footpad (MFP5). No difference in gene expression levels were observed between ML48 and ML96.

Supplementary Table S1 Formulation of NHDP axenic medium for *in vitro* maintenance of *M. leprae*.

Supplementary Table S2 Potential homologs for twelve highly expressed *M. leprae* hypothetical proteins.
