# Supplementary figures and images for: Mycobacterium leprae Transcriptome During In Vivo Growth and Ex Vivo Stationary Phases
Source: Front Cell Infect Microbiol. 2022 Jan 12;11:817221. doi: 10.3389/fcimb.2021.817221 (PMC8790229; doi:10.3389/fcimb.2021.817221)

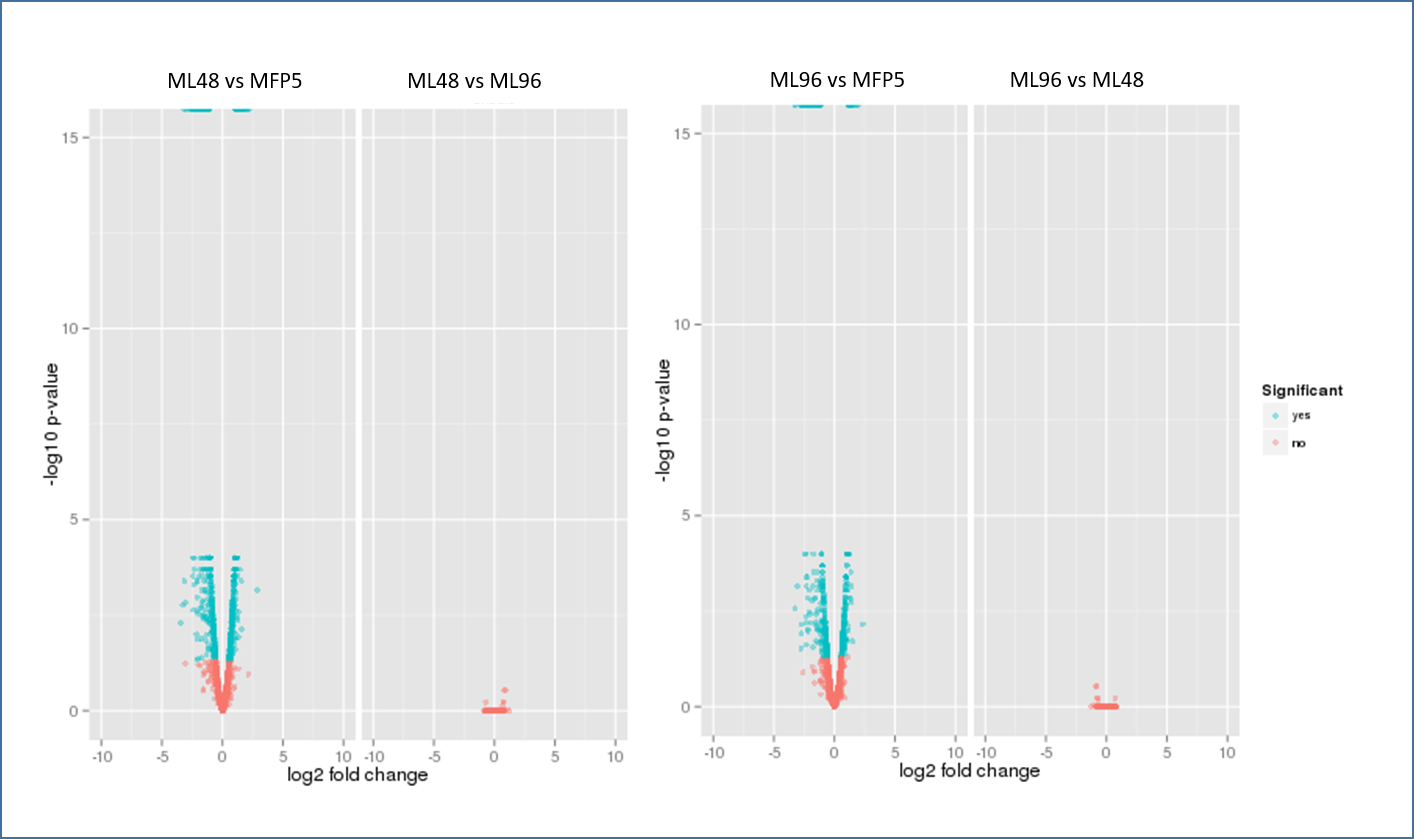

Supplement: Supplementary Figure S1 — Volcano plot showing significant changes in gene expression in ML48 and ML96 when compared to freshly harvested M. leprae from mouse footpad (MFP5). No difference in gene expression levels were observed between ML48 and ML96. [file Image_1.tif]
