# Supplementary material for: Mycobacterium leprae Transcriptome During In Vivo Growth and Ex Vivo Stationary Phases
Source: Front Cell Infect Microbiol. 2022 Jan 12;11:817221. doi: 10.3389/fcimb.2021.817221 (PMC8790229; doi:10.3389/fcimb.2021.817221)
Supplement: Supplementary Table S1 — Formulation of NHDP axenic medium for in vitro maintenance of M. leprae. [file Table_1.pdf]

| **Component** | **gm/L** |
| --- | --- |
| **7H9 powder** | 4.68 |
| **Bovine Albumin (Fraction V)** | 5.0 |
| **Dextrose** | 8.0 |
| **Casitone/Casein Hydrolysate** | 1.0 |

**Supplementary Table S1.** Formulation of NHDP axenic medium for *in vitro* maintenance of *M. leprae*.
