# Supplementary material for: Mycobacterium leprae Transcriptome During In Vivo Growth and Ex Vivo Stationary Phases
Source: Front Cell Infect Microbiol. 2022 Jan 12;11:817221. doi: 10.3389/fcimb.2021.817221 (PMC8790229; doi:10.3389/fcimb.2021.817221)
Supplement: Supplementary Table S2 — Potential homologs for twelve highly expressed M. leprae hypothetical proteins. [file Table_2.pdf]

***M. leprae* gene No.**	**Mycobacterial Species**	**Potential Homolog**	**% Amino Acid Identity**
ML0659	*M. lepromatosis*	Hypothetical protein MLPM_0659	50
ML0947	*M. lepromatosis*	Hypothetical protein MLPM_0947	81
ML0959	*M. lepromatosis*	Hypothetical protein MLPM_0959	69
ML1275	*M. lepromatosis*	Hypothetical protein MLPM_1275	75
ML1018	*M. lepromatosis*	Hypothetical protein MLMP_1018	52
ML1989	*M. haemophilum*	Hypothetical protein	71
ML0473	*M. haemophilum*	NIFU family protein	77
ML1763	*M. haemophilum*	Potassium transporter TrkA	69
ML1796	*M. intracellulare*	transcription anti-termination regulator	60
ML2252	Multiple species	DivIVA, cell division protein	53
ML0678	Multiple species	IS110 transposase	63
ML0023	Multiple species	IS481 family mycobacterial transposases	67

Supplementary Table S2. Potential homologs for twelve highly expressed *M. leprae* hypothetical proteins.
